# Supplementary material for: Lymphocyte innateness defined by transcriptional states reflects a balance between proliferation and effector functions
Source: Nat Commun. 2019 Feb 8;10:687. doi: 10.1038/s41467-019-08604-4 (PMC6368609; doi:10.1038/s41467-019-08604-4)
Supplement: Supplementary file 1 — Supplementary Information [file 41467_2019_8604_MOESM1_ESM.pdf]

Supplementary Information

Lymphocyte innateness defined by transcriptional states reflects a balance between proliferation and effector functions

Maria Gutierrez-Arcelus[1,2,3,4], Nikola Teslovich[1,2,3], Alex R. Mola[3], Rafael B. Polidoro[3], Aparna Nathan[1,2,3,4], Hyun Kim[1,2,3], Susan Hannes[1,2,3,4], Kamil Slowikowski[1,2,3,4], Gerald F. M. Watts[3], Ilya Korsunsky[1,2,3,4], Michael B. Brenner[3], Soumya Raychaudhuri*[,1,2,3,4,5], Patrick J. Brennan*[,3]

Affiliations:

[1]Department of Medicine, Division of Genetics, Brigham and Women's Hospital, Harvard Medical School, Boston, MA, USA 02115.

[2]Program in Medical and Population Genetics, Broad Institute, Cambridge, MA 02142, USA.

[3]Department of Medicine, Division of Rheumatology, Immunology and Allergy, Brigham and Women's Hospital, Harvard Medical School, Boston, MA 02115, USA.

[4]Center for Data Sciences, Brigham and Women's Hospital, Harvard Medical School, Boston, MA 02115, USA.

[5]Faculty of Medical and Human Sciences, University of Manchester, Manchester M13 9PL, UK.

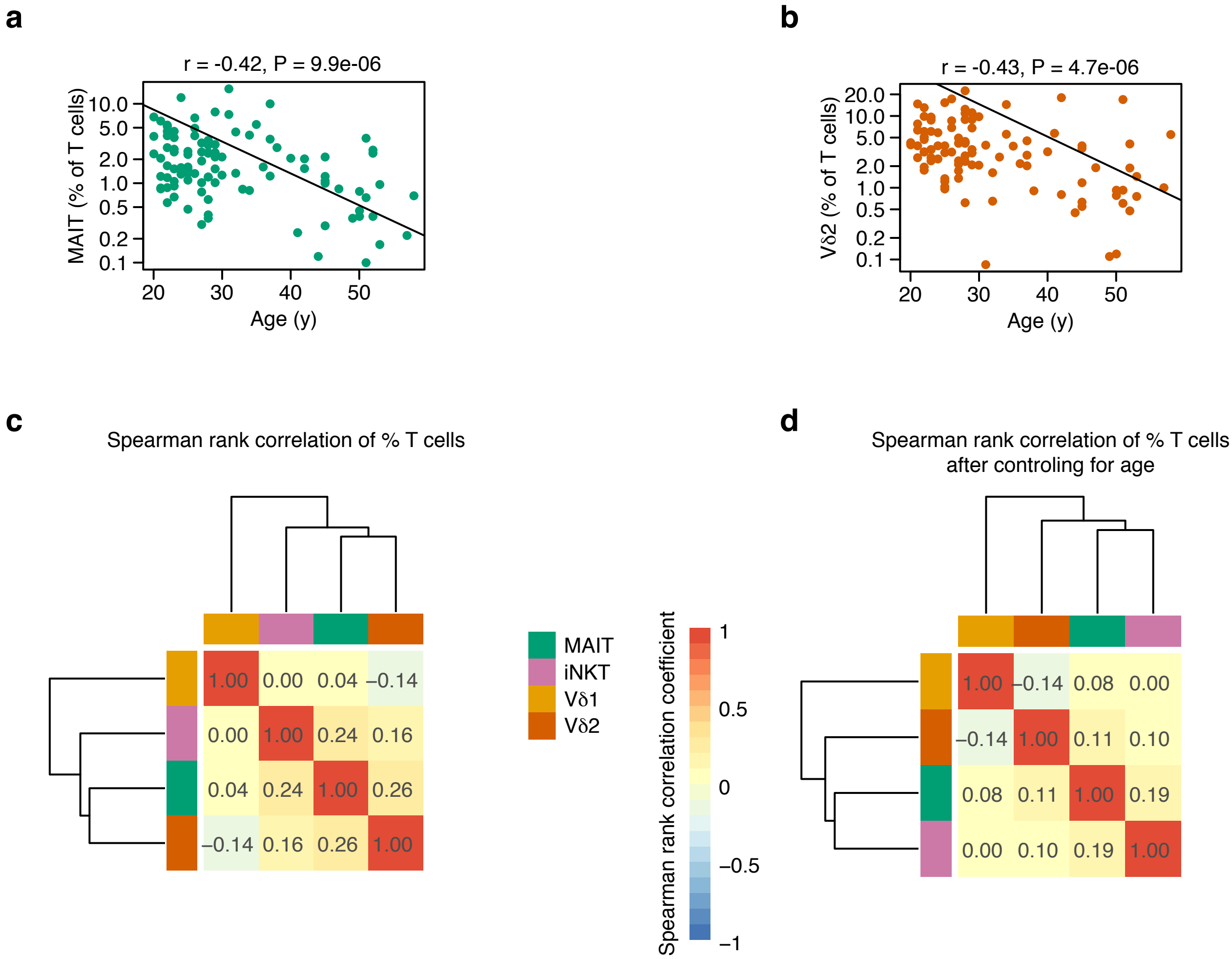

Supplementary Figure 1

Innate T cell population frequency associations with age and covariance.

Associations between donor age and (a) MAIT or (b) Vδ2 T cells, r from Pearson correlations, P-values from t-test. Heatmaps depict pairwise Spearman correlation coefficients between T cell count percentages in 101 healthy individuals, (c) before and (d) after regressing out age effects.

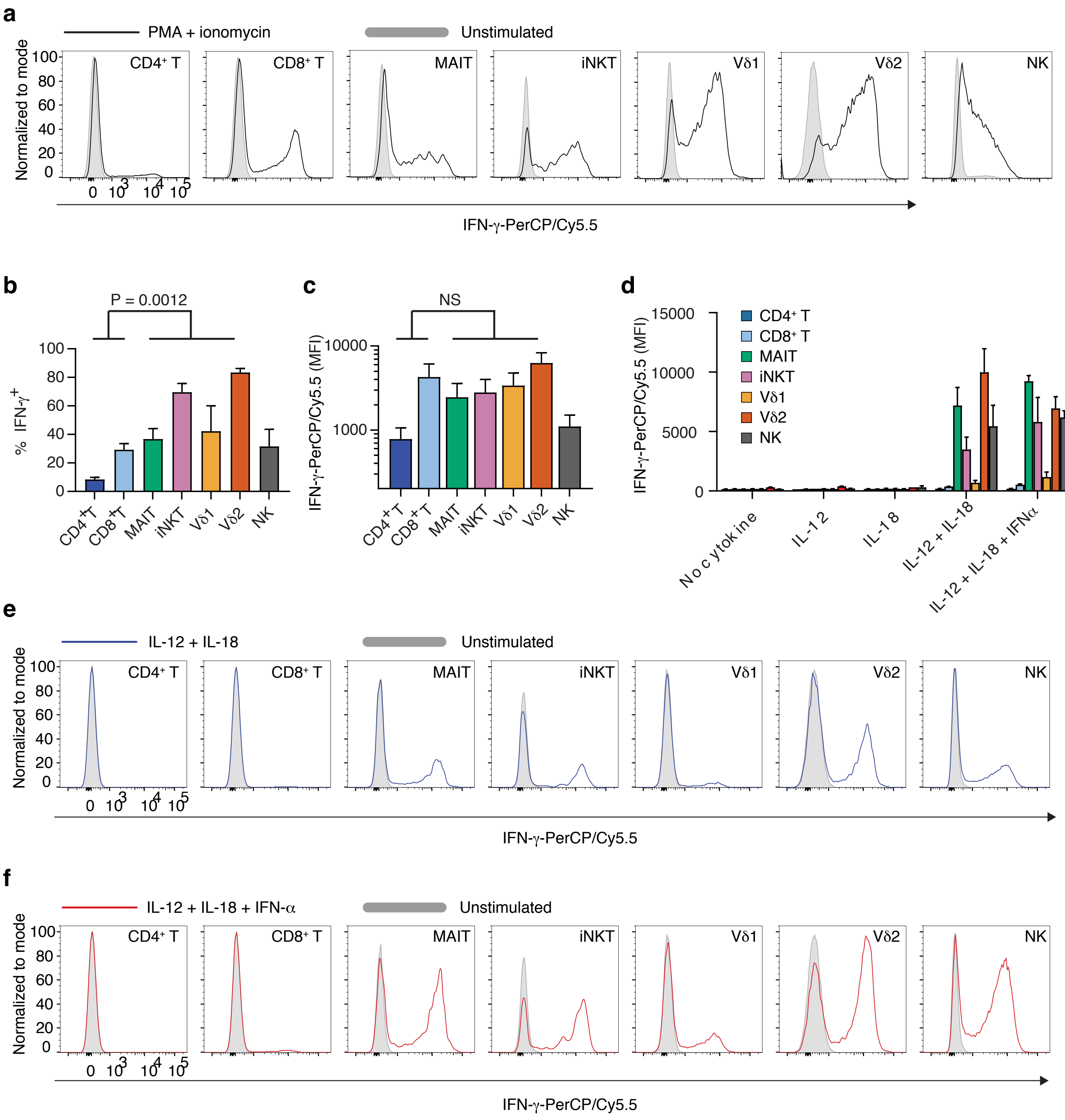

Supplementary Figure 2

Activation of lymphocyte populations.

PBMCs were activated with PMA and ionomycin for 5 hrs and IFN-γ production was quantified by intracellular cytokine staining, (a) a representative plot, and (b,c) N=3, SEM, Mann-Whitney test. (d) PBMCs were activated for 16 hrs with cytokines, with IFN-γ production quantified during the last 4 hrs by intracellular cytokine staining N=3, SEM. (e,f) Representative flow plots following cytokine-only activation.

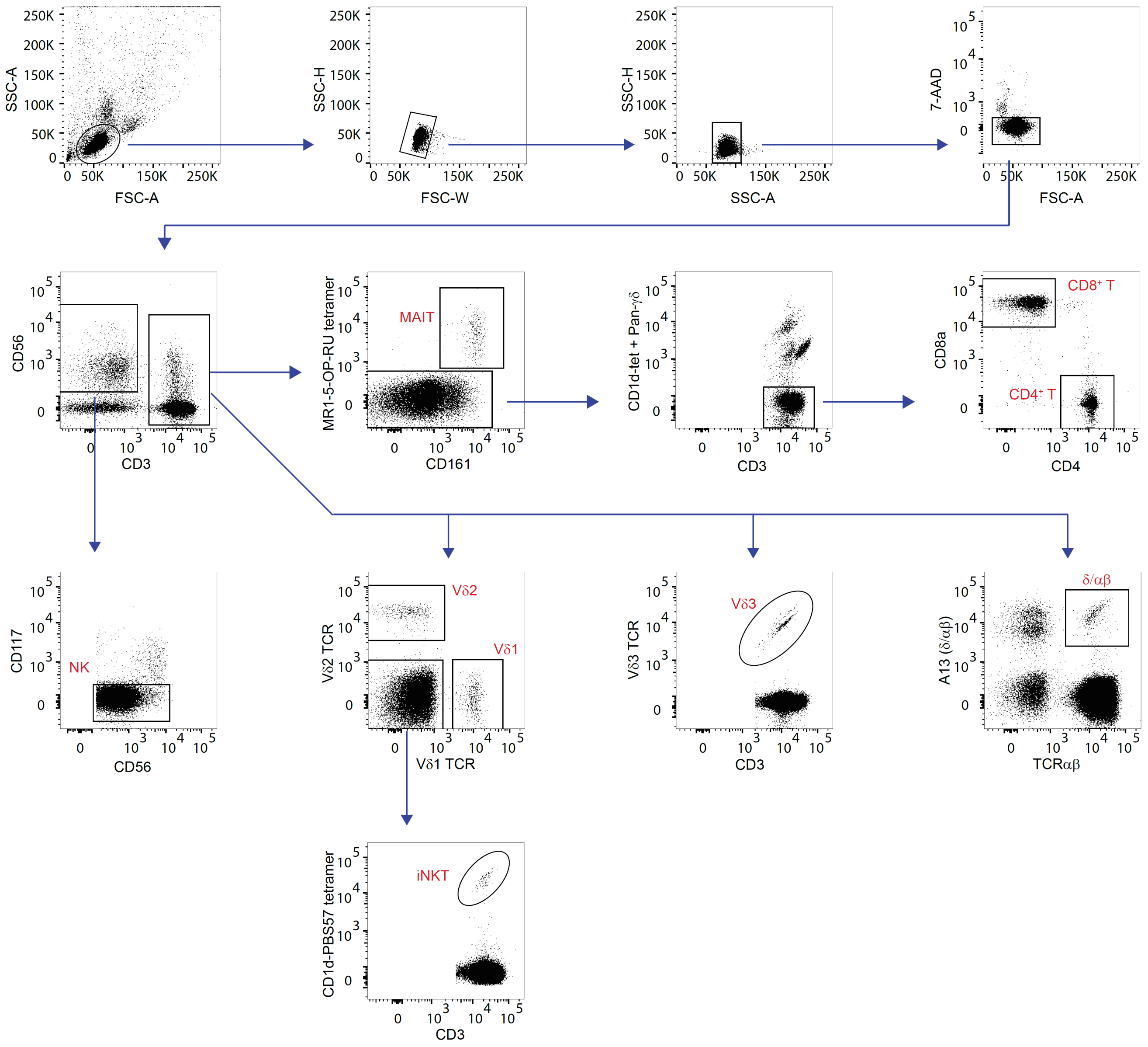

Supplementary Figure 3

Gating strategy used for sorting for RNA-seq and validation experiments.

For validation studies, MR1-5-OP-RU tetramer was replaced by anti-Vα7.2 TCR.

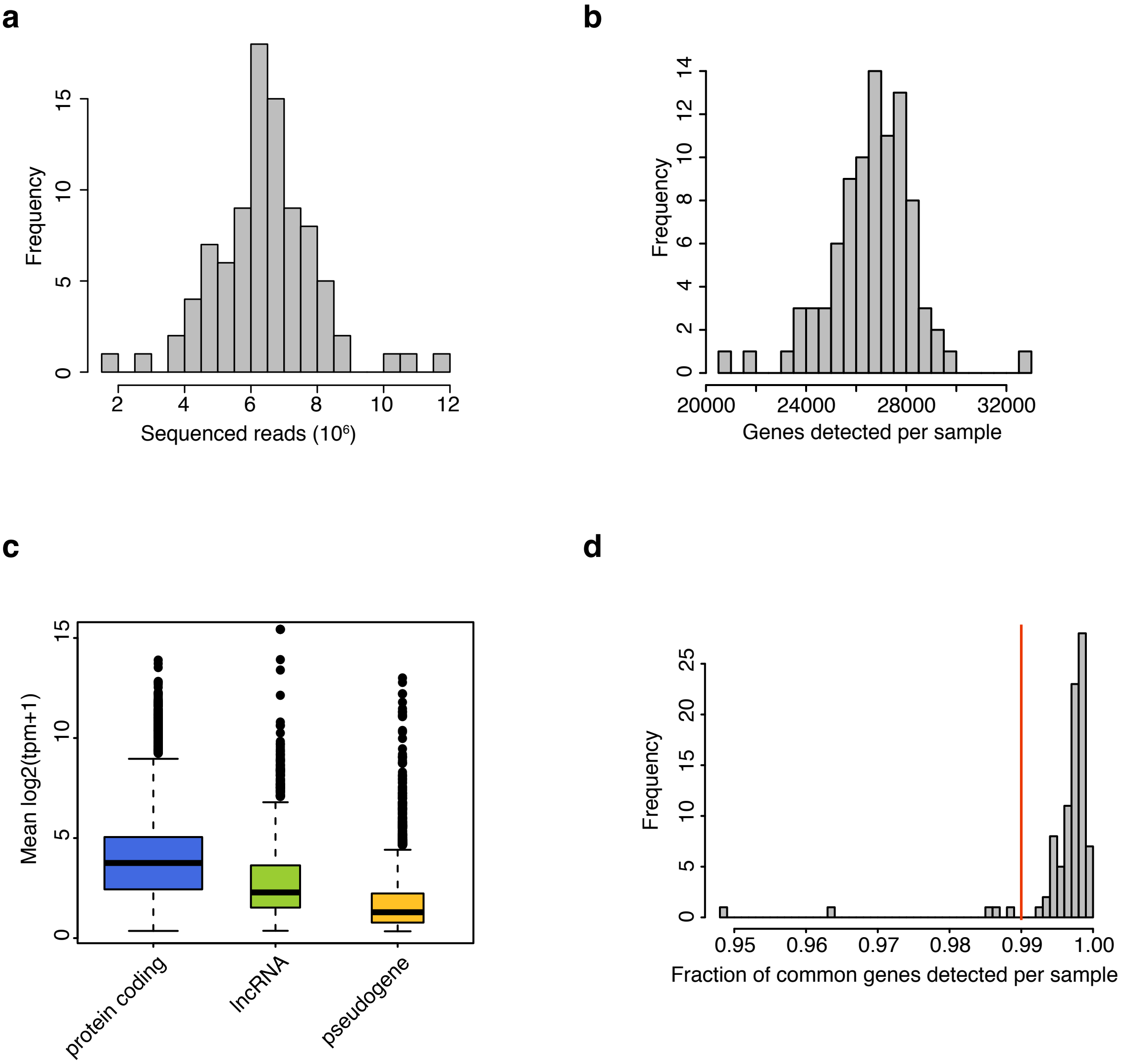

Supplementary Figure 4

RNA-seq summary and quality check.

(a) Number of sequenced reads, and (b) number of genes detected per sample. (c) Distributions of mean expression levels for protein-coding genes, lncRNA genes, and pseudogenes. (d) Fraction of common genes detected per sample. Samples to the left of the vertical red line were considered low quality and were discarded from further analyses.

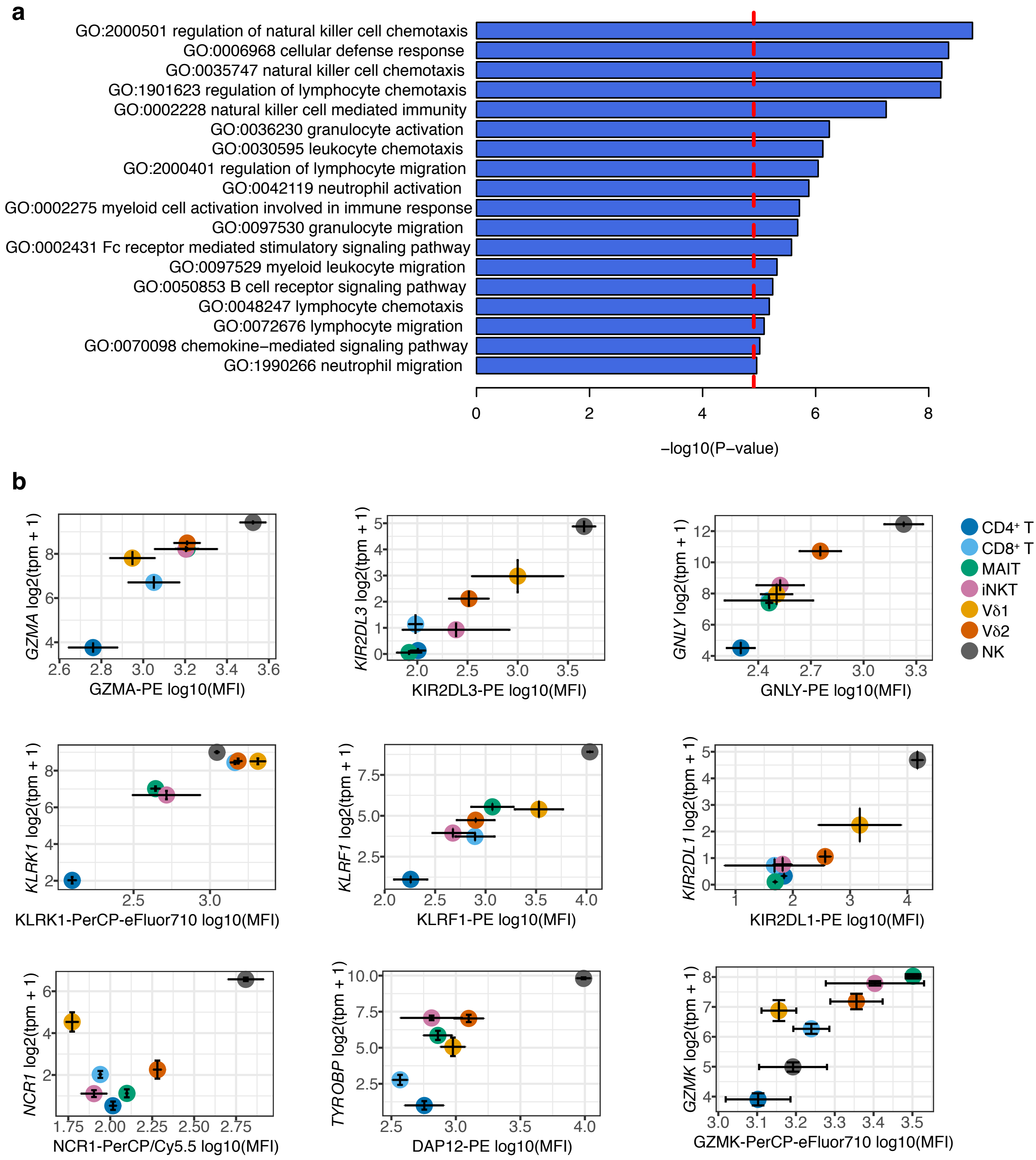

Supplementary Figure 5

Innateness-associated genes and pathways.

(a) GO terms significantly associated within innateness genes, hypergeometric test. Dashed red line indicates Bonferroni threshold (0.05 divided by number of tests). (b) Flow cytometric validation for innateness associated genes, showing protein levels by flow cytometry (X-axis), and transcript levels with RNA-seq (Y-axis). tpm, N=6; MFI, N=3.

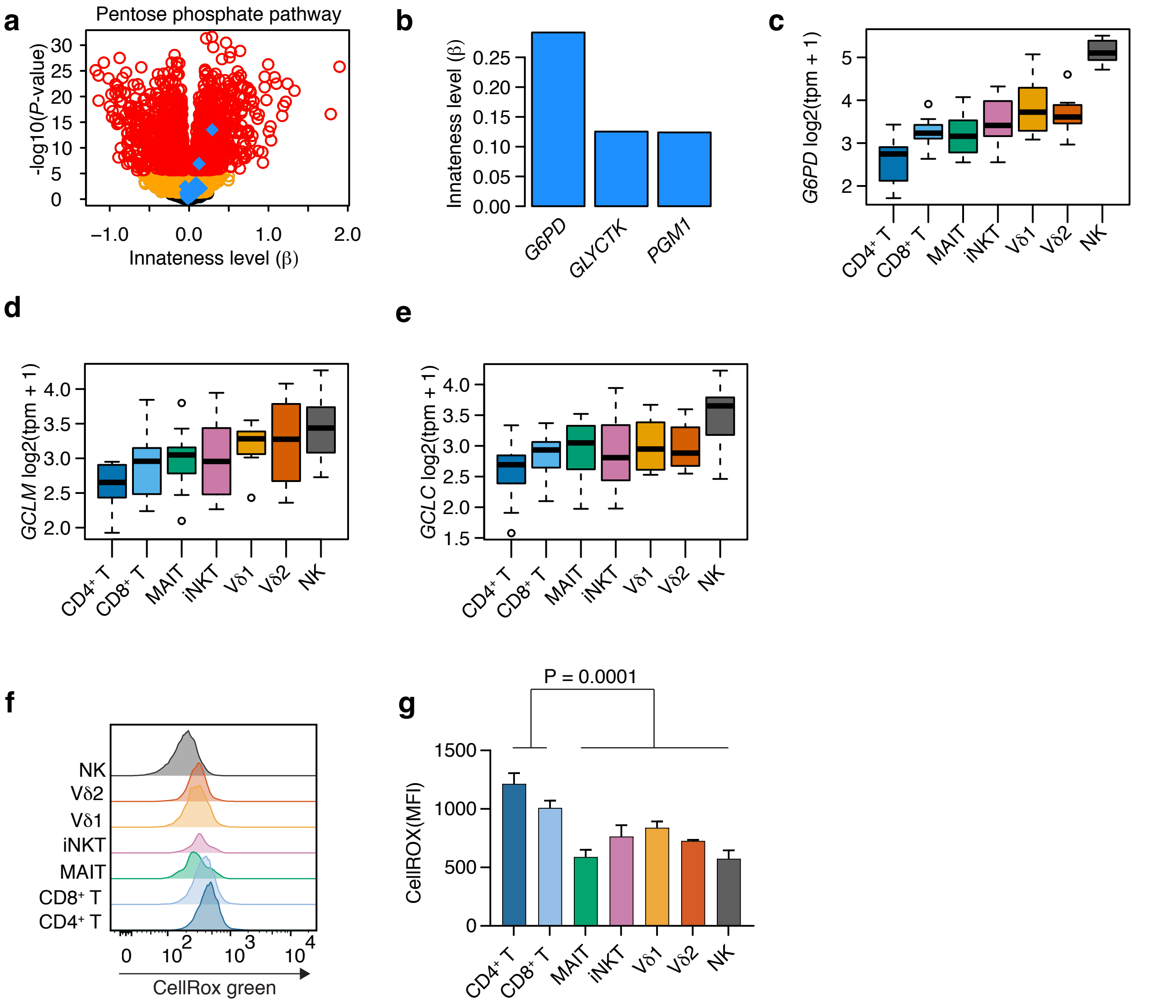

Supplementary Figure 6

Elevated G6PD and lower ROS are associated with innateness.

(a) Volcano plot showing associations with innateness gradient. Yellow, genes with $P < 0.05$; red, genes with $P < 2.5e\text{-}06$ (Bonferroni threshold); blue, KEGG pentose phosphate pathway (hsa00030). (b) Innateness level (β) for genes in pentose phosphate pathway with $P < 2.5e\text{-}06$. Distribution by cell type for transcript levels of (c) G6PD, (d) GCLM, and (e) GCLC. (f) A representative plot (normalized to mode) and (g) N=3 with s.e.m., Mann-Whitney test. PBMCs labeled with CellRox green for one hour to quantify total cellular ROS, followed staining with markers to identify ITC populations. Boxplots are described in methods.

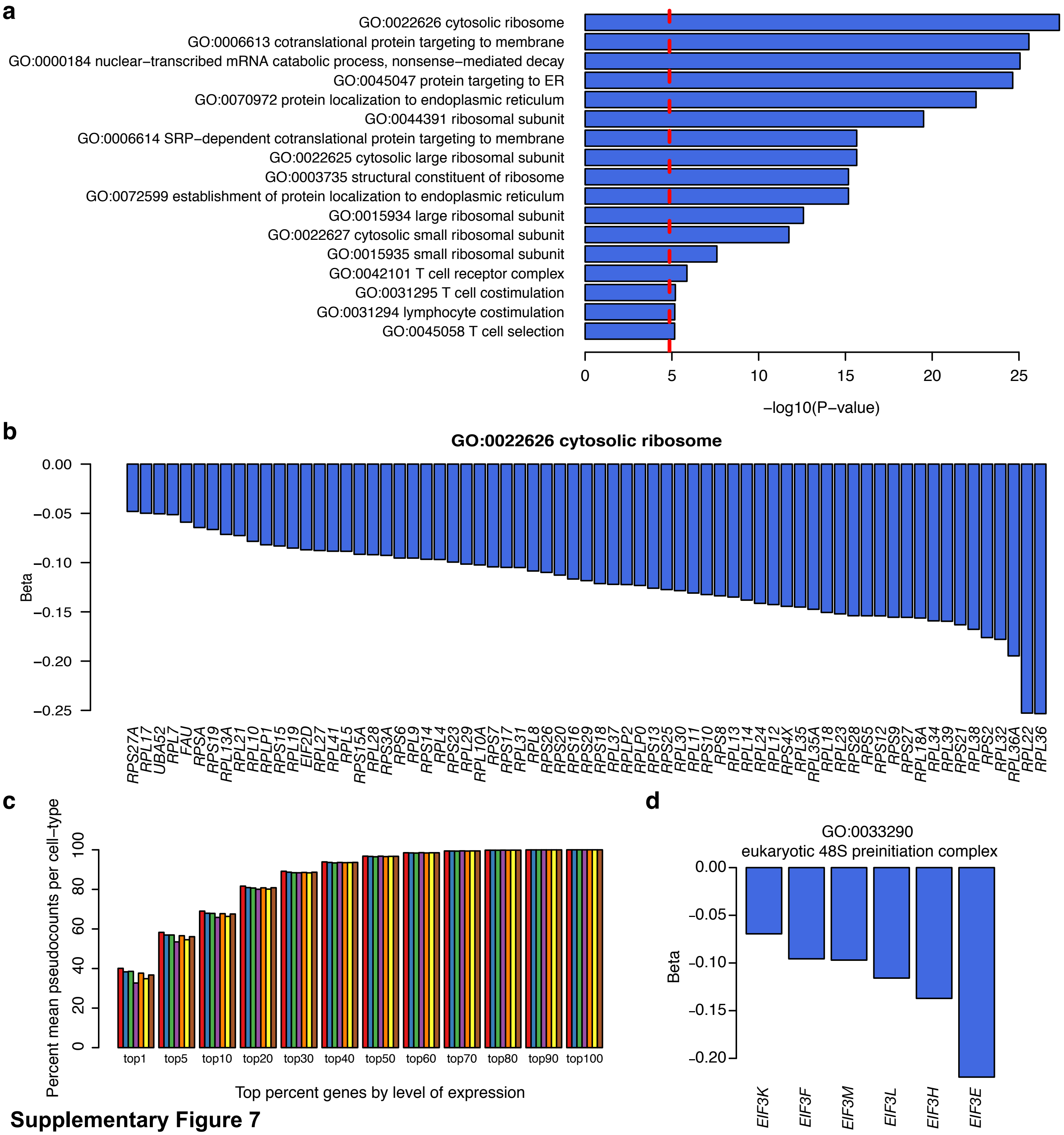

Supplementary Figure 7

Ribosome associations with adaptiveness.

(a) GO terms significantly enriched within adaptiveness genes, hypergeometric test. Dashed red line indicates Bonferroni threshold (0.05 divided by number of tests). (b) Innateness level (β) for genes with GO term cytosolic ribosome (GO:0022626) and with $P < 2.5e{-}06$. (c) Mean percent expression (Y-axis) occupied by the top X% expressed genes (X-axis) per cell-type. Cell types, from left to right are $CD4^+$ T (red), $CD8^+$ T (blue), MAIT (green), iNKT (purple), Vδ1 (orange), Vδ1 (yellow), NK (brown). (d) Innateness level (β) for genes with GO term eukaryotic 48S preinitiation complex (GO:0033290) and with $P < 2.5e{-}06$.

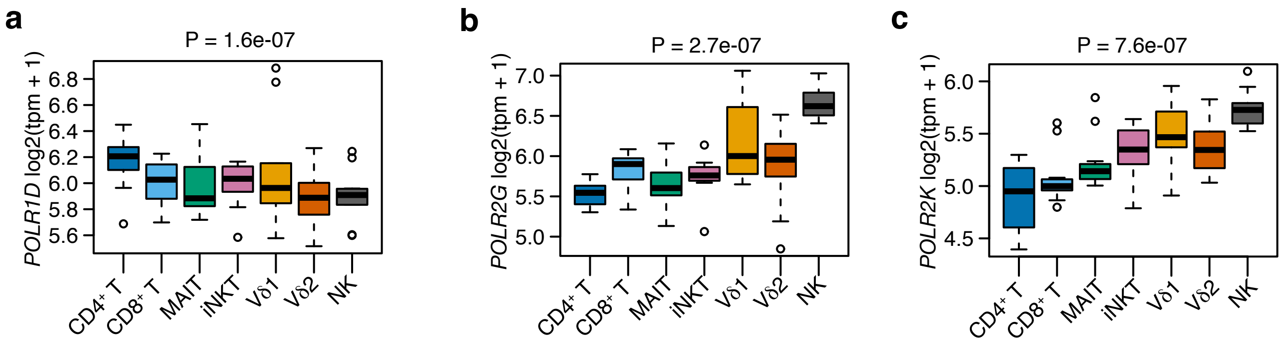

Supplementary Figure 8

Polymerase subunits associated with adaptiveness or innateness.

Distribution by cell type for transcript levels of (a) polymerase I subunit *POLR1D*, that transcribes rRNA, (b-c) polymerase II subunits *POLR2G* and *POLR2K* that transcribe mRNA. N=6 donors, 1-2 replicates per cell type. Boxplots are described in methods. P-values are from associations with innateness.

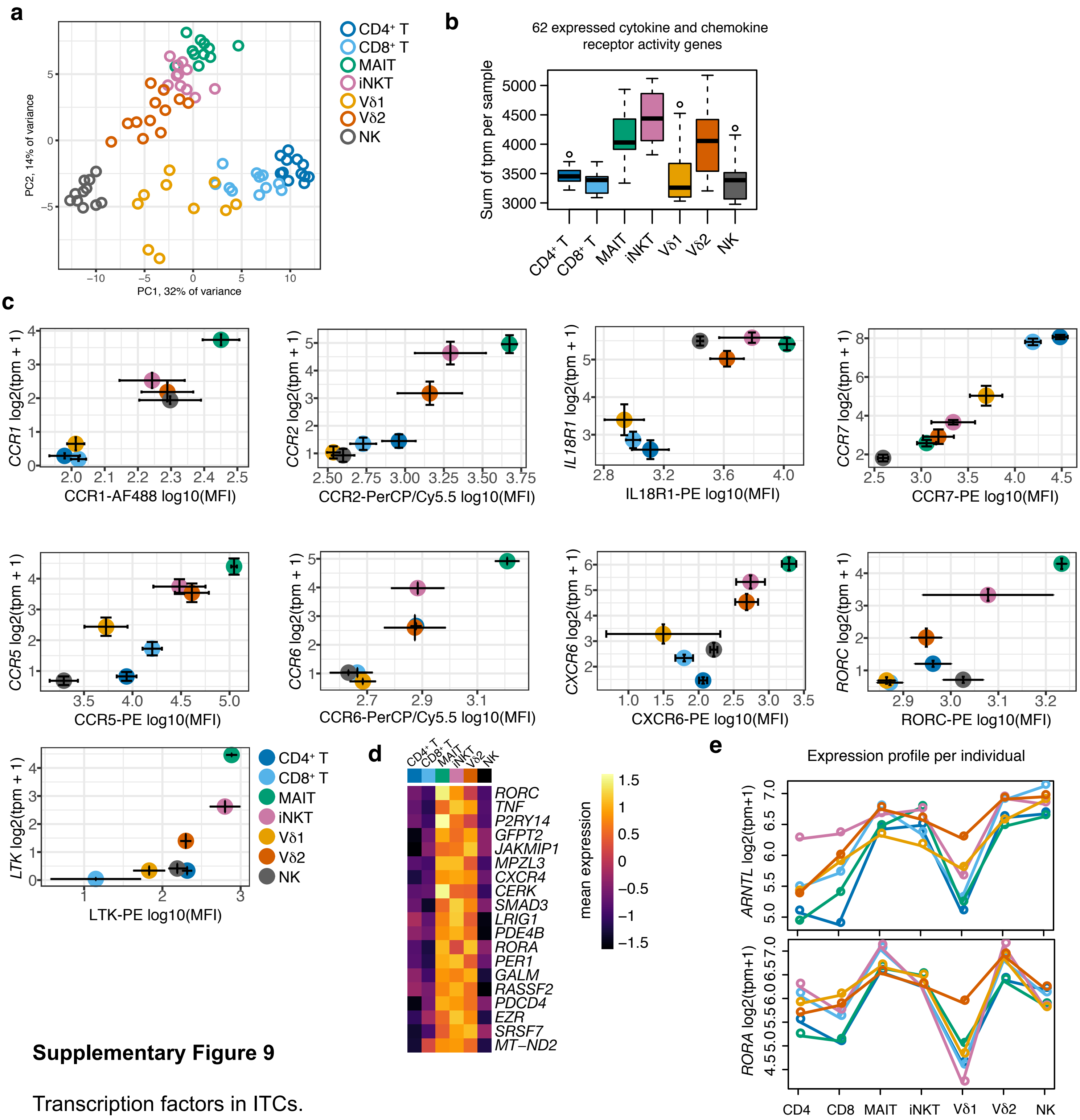

Supplementary Figure 9

Transcription factors in ITCs.

(a) Principal component analysis performed on 142 transcription factor genes variable among cell types (F statistic, P < 5.7e-05, Bonferroni threshold), centered and scaled genes. Plotted are scores for PC1 and PC2. (b) Sum of expression levels for 62 cytokine and chemokine receptor genes across samples. (c) Flow cytometric validation for genes differentially expressed between PLZF⁺ ITCs and adaptive T cells, showing protein levels by flow cytometry (X-axis), and transcript levels with RNA-seq (Y-axis). tpm, N=6; MFI, N=3. (d) Heatmap for mean expression level of PLZF target genes in mouse. Genes shown are upregulated in PLZF⁺ ITCs compared to adaptive T cells and low in NK cells. (e) Mean expression per individual (colored lines) among different cell types for circadian transcription factors ARNTL (that codes for BMAL, top) and RORA (bottom). All cell isolations were performed in the morning.

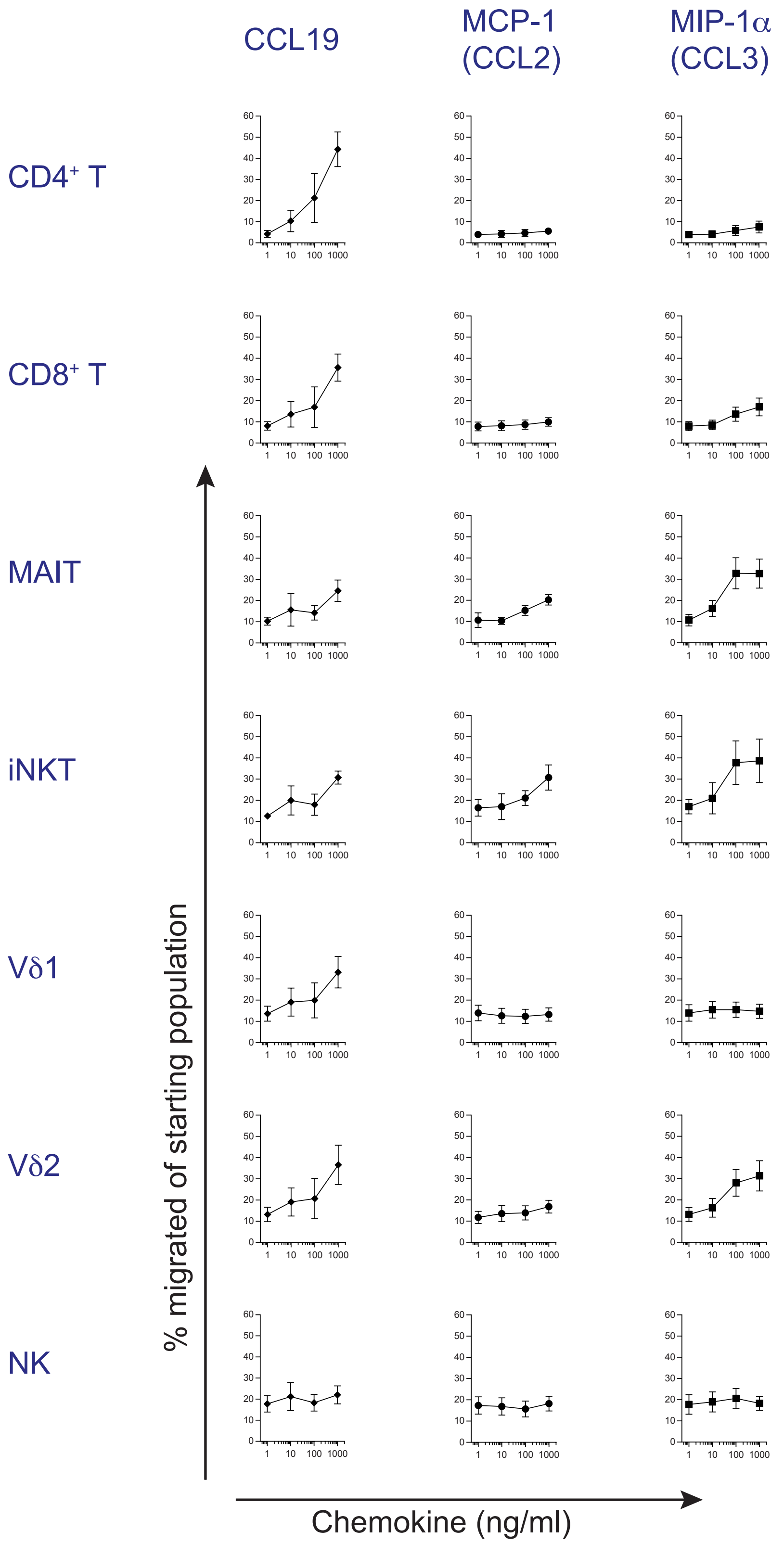

Supplementary Figure 10

ITCs migrate toward 'innate' chemokines.

PMBCs were exposed to chemokines through a semi-permeable membrane (Transwell), and the percentage of cells migrating through the membrane was assessed after 3 hrs. N=3 independent donors, error is s.e.m.

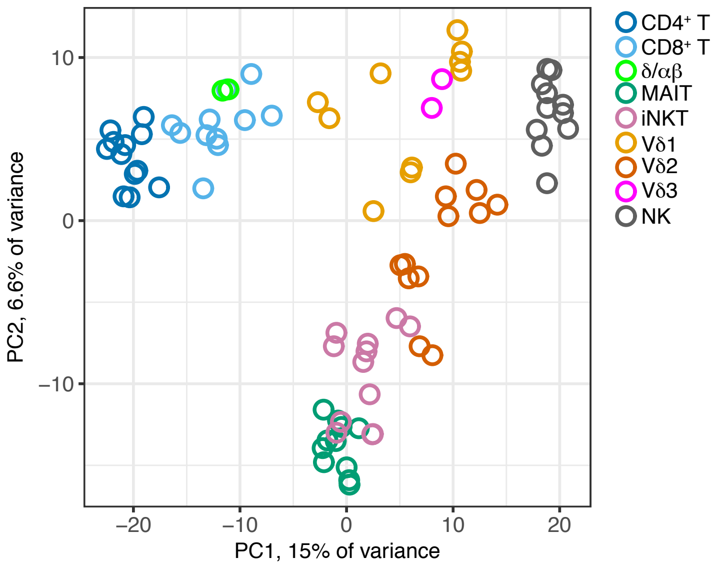

Supplementary Figure 11

Innateness in candidate ITCs.

Principal component analysis including δα/β and Vδ3 T cells, performed on the top 1,012 variable and expressed genes, centered and scaled. Plotted are scores for PC1, PC2.

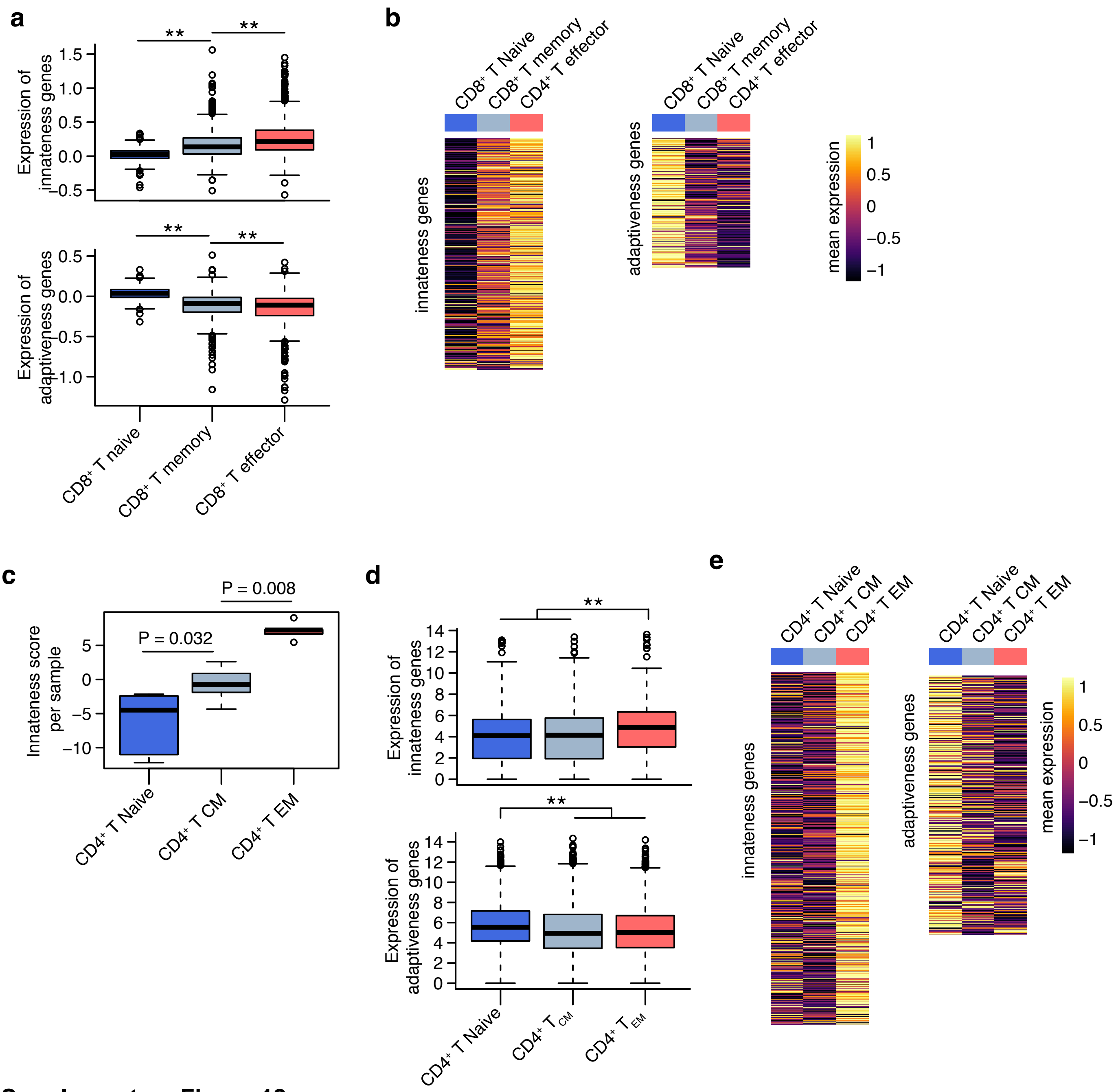

Supplementary Figure 12

Innateness gradient genes in adaptive T cells.

Innateness gradient genes in (a-b) HCMV-specific $CD8^+$ T cell populations (naive = $CD45RA^+CD27^{bright}$; memory = $CD45RA^-CD27^+$; effector = $CD45RA^+CD27^-$), and (c-e) $CD4^+$ T cell populations (naive = $CCR7^+CD45RA^+CD45RO^-$; T central memory (T_{CM}) = $CCR7^+CD45RA^-CD45RO^+$; T effector memory (T_{EM}) = $CCR7^-CD45RA^-CD45RO^+$). (a,d) Depicted are distributions of mean expression levels for innateness genes (upper) and adaptiveness genes (lower), averaged across 4 replicates for (a) and 5 replicates for (d). ** $P < 3e-11$, Wilcoxon paired test. (b,e) Heatplots of mean expression per cell-type for genes associated with innateness (left) or adaptiveness (right), for (b) HCMV-specific $CD8^+$ T cells and (e) $CD4^+$ T cells. (c) Innateness score per sample. N=5 replicates per cell-type. P-values shown for Wilcoxon test. Innateness score and boxplots are described in methods.

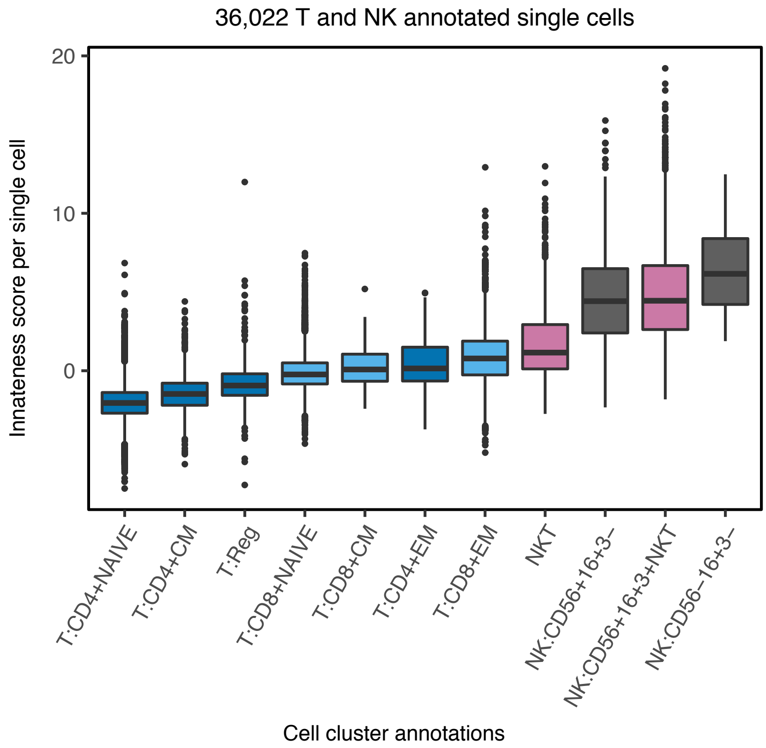

Supplementary Figure 13

Innateness gradient recapitulated for T and NK annotated single cell clusters.

Innateness score was calculated per single cell in cell clusters annotated as T or NK cell types. Cells were isolated from tumor, normal breast tissue, blood, and lymph node of 8 cancer patients.

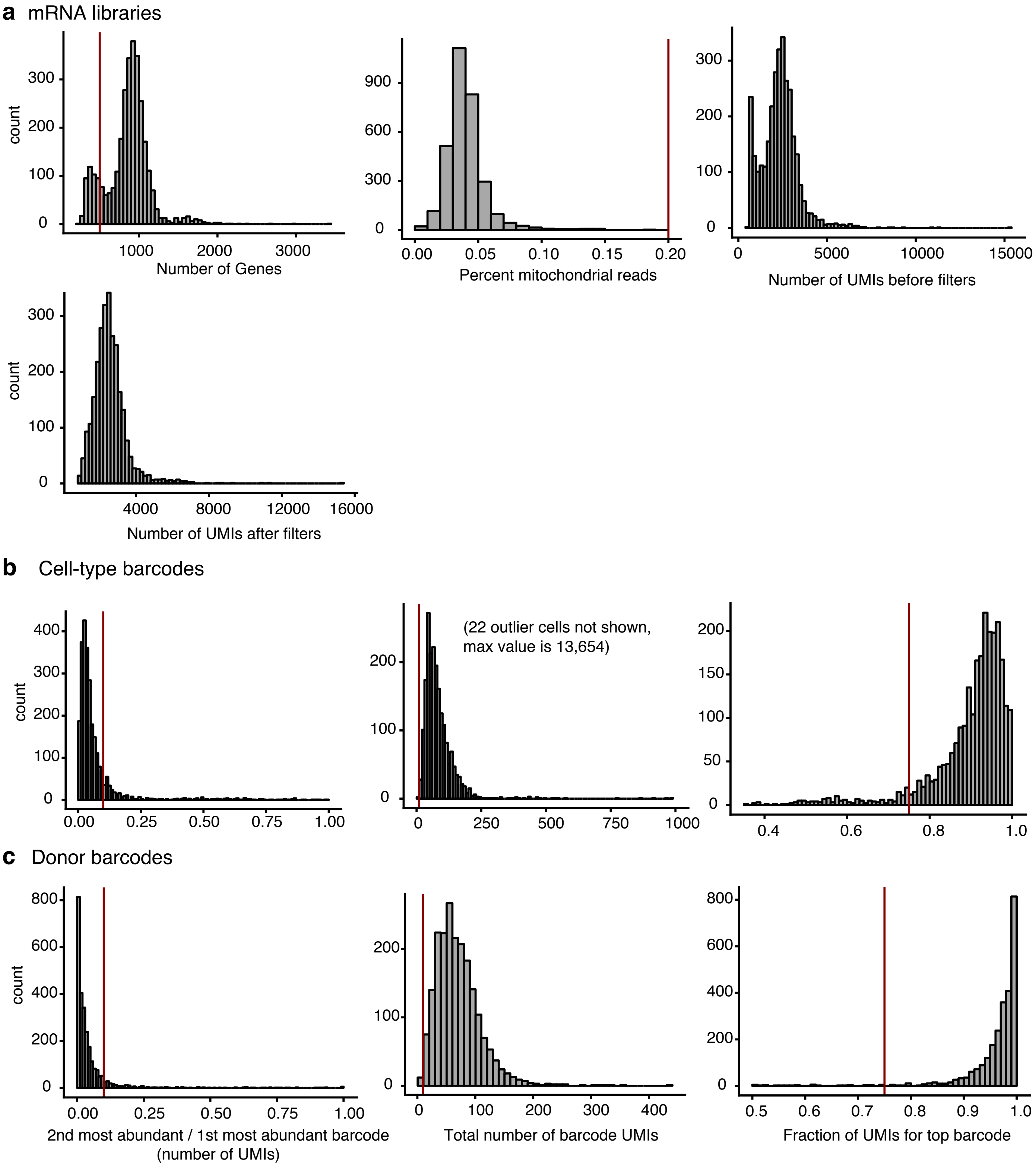

Supplementary Figure 14

Single-cell RNA-seq data metrics and quality control.

Distributions of metrics for (a) single-cell in mRNA libraries, and (b) hashing antibody libraries for (b) cell-type barcodes and (c) donor barcodes. Red lines indicate thresholds applied for filtering each type of data as detailed in methods.

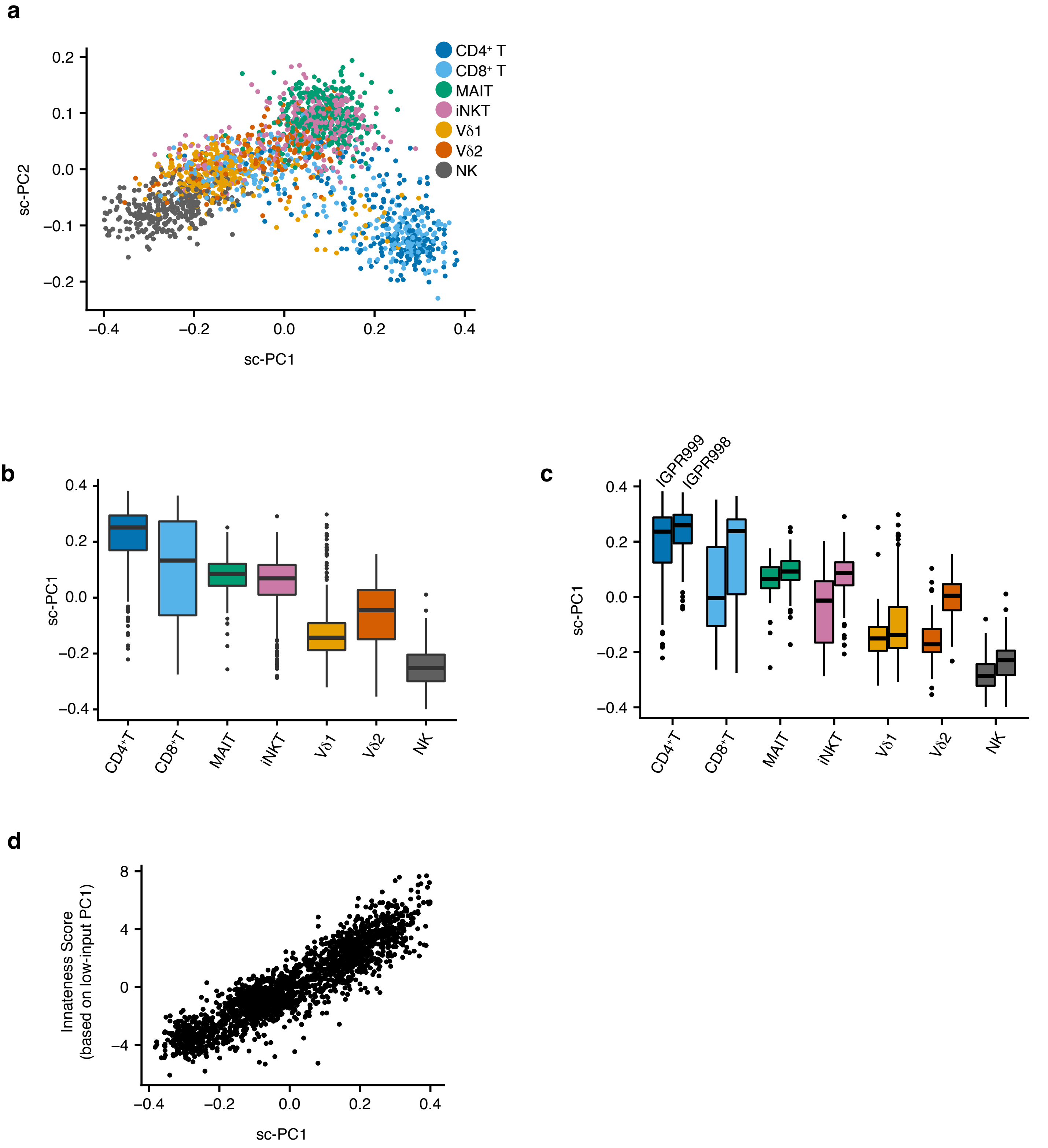

Supplementary Figure 15

Single-cell RNA-seq dimensionality reduction.

(a) PCA on top 1,545 most variable genes in single-cell RNA-seq. (b) sc-PC1 scores by cell-type. (c) sc-PC1 scores, within each cell-type left and right boxplots correspond to donor IGPR999 and IGPR998, respectively. (d) sc-PC1 is correlated with innateness score per cell (based on low-input PC1).

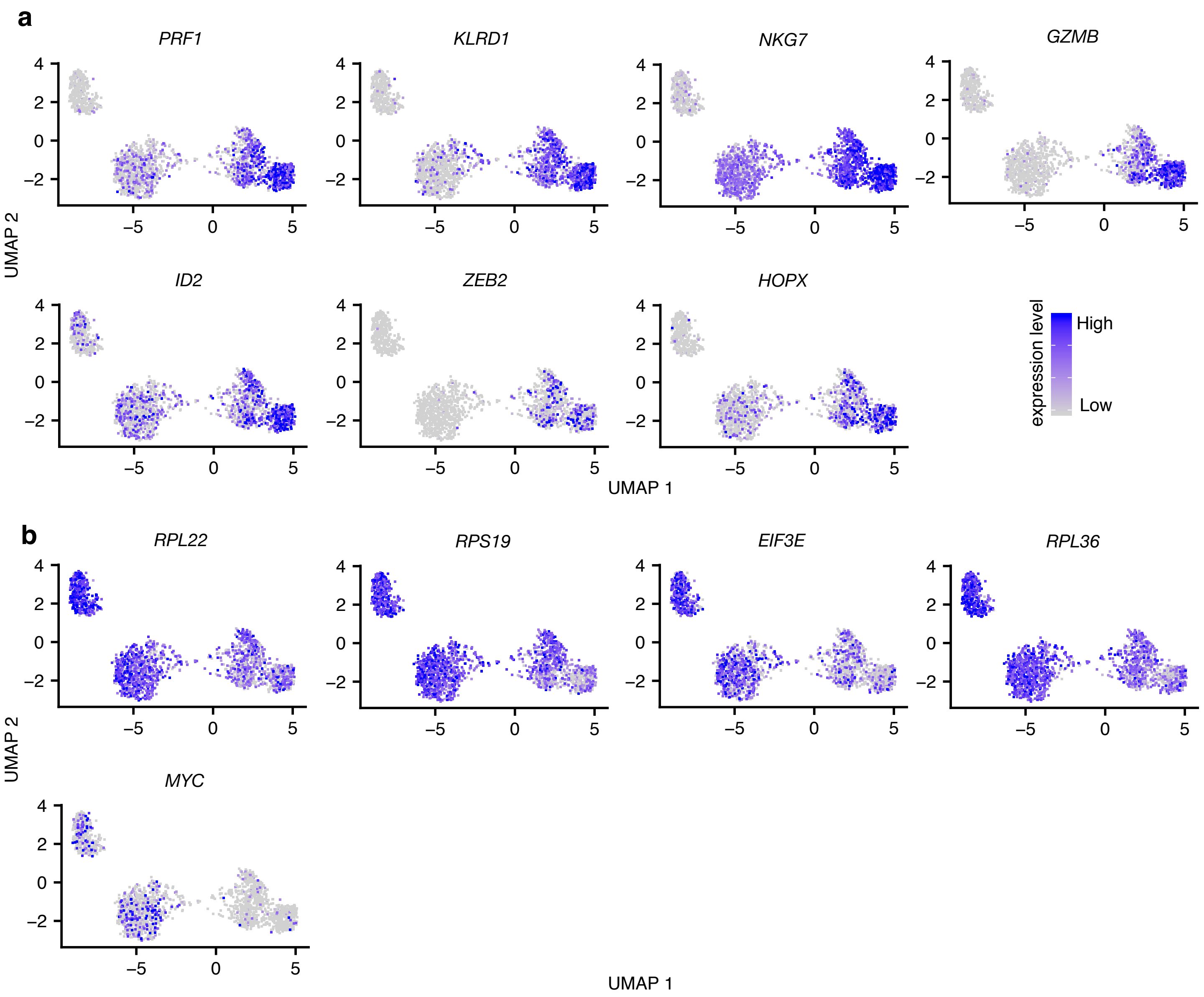

Supplementary Figure 16

Expression of innateness and adaptiveness markers in single cells.

Single cells over UMAP space colored by normalized expression levels of (a) innateness genes, and (b) adaptiveness genes.

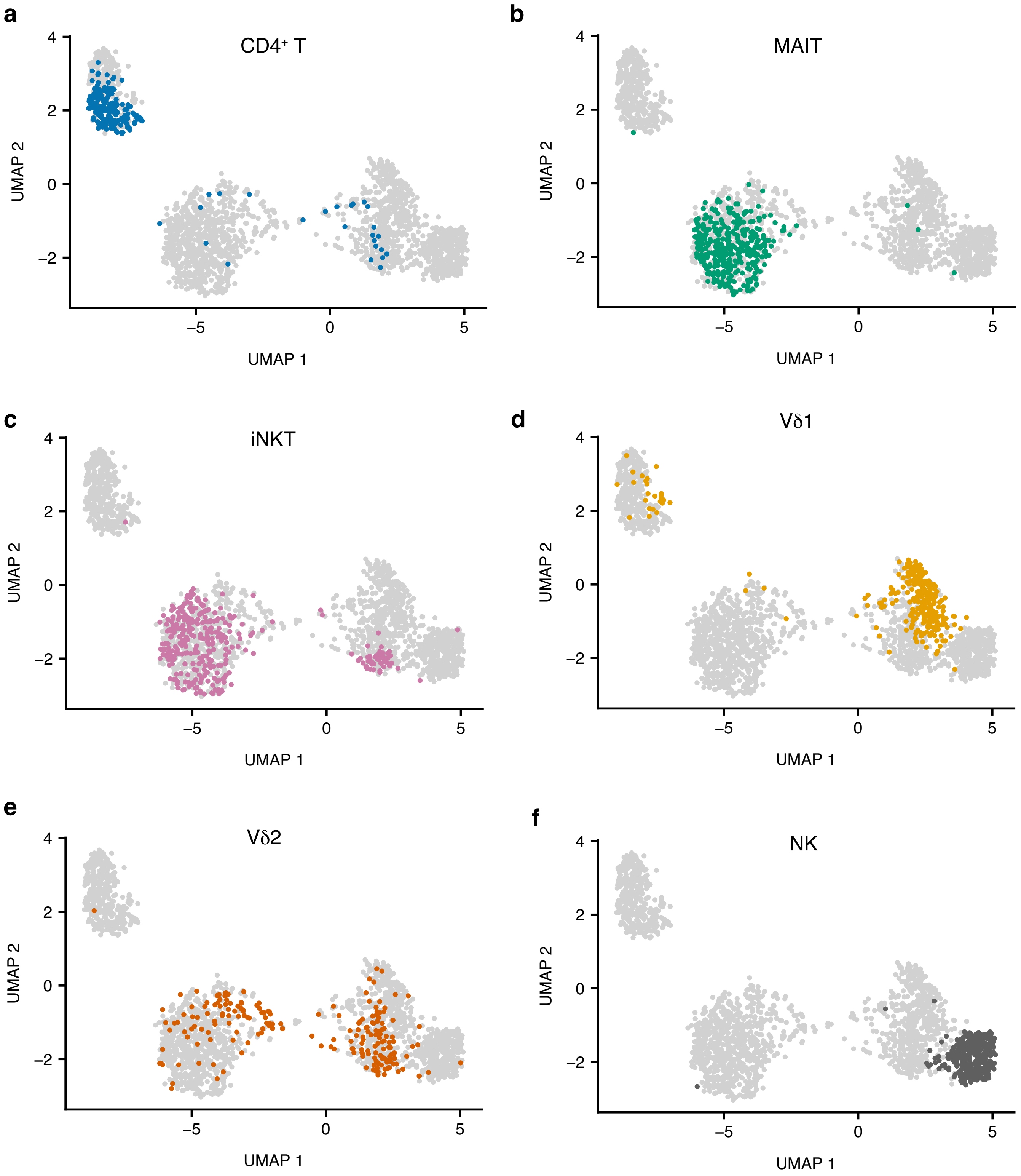

Supplementary Figure 17

Heterogeneity within cell types.

Single cells in UMAP space colored by cell type based on the hashing antibody barcode.

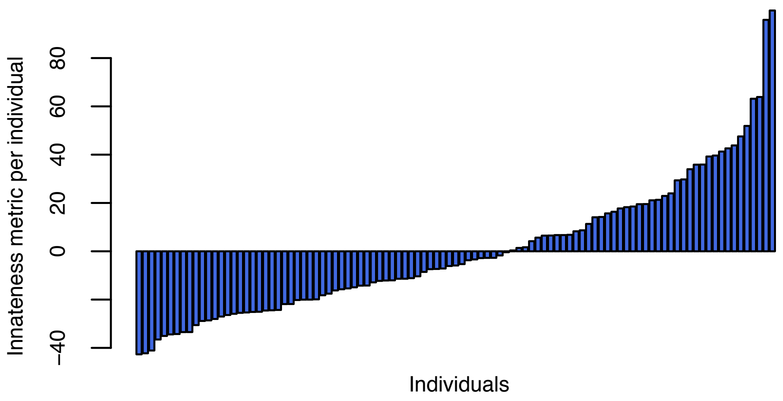

Supplementary Figure 18

Individual innateness metric.

Innateness metric calculated per individual by integrating the immunoprofiling data with the innateness gradient rank per cell type. Specifically, we summed the abundance per cell type (proportion of T cells) multiplied by the rank of that cell type in the innateness gradient. We then regressed out the age effects. Plotted are the residuals of this regression.

Supplementary Table 1 T cell subset samples isolated and RNA-sequenced.

Subset	Cells per Sample	Samples post-QC	Individuals post-QC
$CD4^+$ T	1000	12	6
$CD8^+$ T	1000	11	6
MAIT	1000	11	6
iNKT	1000	12	6
Vδ1	1000	10	6
Vδ2	1000	12	6
NK	1000	11	6
Vδ3	1000	2	1
δ/αβ	1000	2	1

Supplementary Table 2 Number of cells that passed quality check.

**Subset**	**Donor 1**	**Donor 2**	**Total**
$CD4^+$ T	101	121	222
$CD8^+$ T	135	175	310
MAIT	139	243	382
iNKT	98	211	309
Vδ1	86	239	325
Vδ2	130	154	284
NK	70	134	204
**Total**	759	1277	2036
