# Supplementary material for: Lymphocyte innateness defined by transcriptional states reflects a balance between proliferation and effector functions
Source: Nat Commun. 2019 Feb 8;10:687. doi: 10.1038/s41467-019-08604-4 (PMC6368609; doi:10.1038/s41467-019-08604-4)
Supplement: Supplementary file 3 — Description of Additional Supplementary Files [file 41467_2019_8604_MOESM3_ESM.pdf]

Description of Additional Supplementary Files

File Name: Supplementary Data 1
Description: T cell subset abundances and clinical information of 101 individuals.

File Name: Supplementary Data 2
Description: RNA-seq QC metrics per sample.

File Name: Supplementary Data 3
Description: Results of gene expression associations with innateness gradient in low input RNA-seq.

File Name: Supplementary Data 4
Description: Antibody information.
