# Supplementary material for: Lymphocyte innateness defined by transcriptional states reflects a balance between proliferation and effector functions
Source: Nat Commun. 2019 Feb 8;10:687. doi: 10.1038/s41467-019-08604-4 (PMC6368609; doi:10.1038/s41467-019-08604-4)
Supplement: Supplementary file 8 — Reporting Summary [file 41467_2019_8604_MOESM8_ESM.pdf]

nature research

Corresponding author(s): Patrick Brennan
Soumya Raychaudhuri

Reporting Summary

Nature Research wishes to improve the reproducibility of the work that we publish. This form provides structure for consistency and transparency in reporting. For further information on Nature Research policies, see Authors & Referees and the Editorial Policy Checklist.

Statistical parameters

When statistical analyses are reported, confirm that the following items are present in the relevant location (e.g. figure legend, table legend, main text, or Methods section).

n/a	Confirmed	
☐	☒	The exact sample size (n) for each experimental group/condition, given as a discrete number and unit of measurement
☐	☒	An indication of whether measurements were taken from distinct samples or whether the same sample was measured repeatedly
☐	☒	The statistical test(s) used AND whether they are one- or two-sided<br>*Only common tests should be described solely by name; describe more complex techniques in the Methods section.*
☐	☒	A description of all covariates tested
☐	☒	A description of any assumptions or corrections, such as tests of normality and adjustment for multiple comparisons
☐	☒	A full description of the statistics including central tendency (e.g. means) or other basic estimates (e.g. regression coefficient) AND variation (e.g. standard deviation) or associated estimates of uncertainty (e.g. confidence intervals)
☐	☒	For null hypothesis testing, the test statistic (e.g. F, t, r) with confidence intervals, effect sizes, degrees of freedom and P value noted<br>*Give P values as exact values whenever suitable.*
☒	☐	For Bayesian analysis, information on the choice of priors and Markov chain Monte Carlo settings
☒	☐	For hierarchical and complex designs, identification of the appropriate level for tests and full reporting of outcomes
☐	☒	Estimates of effect sizes (e.g. Cohen's d, Pearson's r), indicating how they were calculated
☐	☒	Clearly defined error bars<br>*State explicitly what error bars represent (e.g. SD, SE, CI)*

Our web collection on statistics for biologists may be useful.

Software and code

Policy information about availability of computer code

Data collection	Methods
Data analysis	Methods

For manuscripts utilizing custom algorithms or software that are central to the research but not yet described in published literature, software must be made available to editors/reviewers upon request. We strongly encourage code deposition in a community repository (e.g. GitHub). See the Nature Research guidelines for submitting code & software for further information.

Data

Policy information about availability of data

All manuscripts must include a data availability statement. This statement should provide the following information, where applicable:

- Accession codes, unique identifiers, or web links for publicly available datasets
- A list of figures that have associated raw data
- A description of any restrictions on data availability

Methods: Data availability

Field-specific reporting

Please select the best fit for your research. If you are not sure, read the appropriate sections before making your selection.

☒ Life sciences ☐ Behavioural & social sciences ☐ Ecological, evolutionary & environmental sciences

For a reference copy of the document with all sections, see nature.com/authors/policies/ReportingSummary-flat.pdf

Life sciences study design

All studies must disclose on these points even when the disclosure is negative.

Sample size	Methods: Study Design
Data exclusions	Methods: Low-input RNA-Seq gene expression quantification, CITE-seq gene expression quantification and cell hashing
Replication	Replications detailed in main text, Figures, Supplementary Figures and Methods.
Randomization	Methods: Study Design
Blinding	N/A

Reporting for specific materials, systems and methods

Materials & experimental systems

n/a	Involved in the study	
☐	☒	Unique biological materials
☐	☒	Antibodies
☒	☐	Eukaryotic cell lines
☒	☐	Palaeontology
☒	☐	Animals and other organisms
☐	☒	Human research participants

Methods

n/a	Involved in the study	
☒	☐	ChIP-seq
☐	☒	Flow cytometry
☒	☐	MRI-based neuroimaging

Unique biological materials

Policy information about availability of materials

Obtaining unique materials	Methods: List of antibodies

Antibodies

Antibodies used	Methods: List of antibodies
Validation	Methods: List of antibodies, manufacturer/supplier information. For unique biological material (A13 antibody), source and references cited.

Human research participants

Policy information about studies involving human research participants

Population characteristics	Supplementary Table S1, Methods
Recruitment	Participants were recruited through the Brigham and Women's Hospital Department of Genetics under an IRB-approved protocol with written consent. These were healthy individuals that were willing to donate blood for a small compensation. This recruitment strategy is highly unlikely to affect our conclusions.

Flow Cytometry

Plots

Confirm that:

☒ The axis labels state the marker and fluorochrome used (e.g. CD4-FITC).

☒ The axis scales are clearly visible. Include numbers along axes only for bottom left plot of group (a 'group' is an analysis of identical markers).

☒ All plots are contour plots with outliers or pseudocolor plots.

☒ A numerical value for number of cells or percentage (with statistics) is provided.

Methodology

Sample preparation	Methods: Flow cytometry and cell sorting
Instrument	Methods: Flow cytometry and cell sorting
Software	Methods: Flow cytometry and cell sorting
Cell population abundance	Fig. 1
Gating strategy	Supplementary Fig. 3, dot plot shown as this format was used for setting gates for cell sorting.

☒ Tick this box to confirm that a figure exemplifying the gating strategy is provided in the Supplementary Information.
